# Risk factors for onset of delirium after neck of femur fracture surgery: a prospective observational study

**DOI:** 10.1051/sicotj/2018018

**Published:** 2018-07-06

**Authors:** Muaaz Tahir, Shahbaz S Malik, Usman Ahmed, Jakub Kozdryk, Syeda Huma Naqvi, Atul Malik

**Affiliations:** 1 The Royal Orthopaedic Hospital NHS Foundation Trust, Birmingham UK; 2 Sandwell and West Birmingham Hospitals NHS Trust, Birmingham UK

**Keywords:** Neck of femur fracture, Rehabilitation, Delirium, Risk factors, Post-operative complications

## Abstract

*Background*: Delirium is a common complication after surgery in the elderly that leads to increased length of stay and other adverse outcomes. The aim of this study was to better understand the exact causes of post-operative delirium in patients undergoing surgery for neck of femur (NOF) fractures.

*Methods*: We performed a prospective cohort study of 381 consecutive patients undergoing surgery for NOF fractures at a single institution. Baseline cognitive status and risk factors were recorded on admission. Post-operative cognitive status was assessed at regular intervals until discharge. Binary logistic regression was performed to identify independent predictors of delirium.

*Results*: Patients who developed post-operative delirium (*n* = 70) were significantly older (average age 83 vs. 78, *p* = 0.019) and more likely to be female (79% vs. 67%, *p* = 0.062) than non-affected patients. The presence of delirium was associated with increased length of stay (13 vs. 10 days, *p* = 0.001) and 1-year mortality (25.7% vs. 15% *p* = 0.03). Independent predictors of delirium included age ≥65 years (Odds Ratio = 5.8), presence of anaemia (OR = 2.9), hypoxia (OR = 2.86), cardiac disease (OR = 2.8), Chronic Obstructive Pulmonary Disease (OR = 2.5), new onset electrolyte imbalance (OR = 2.2) and renal failure (OR = 1.9).

*Conclusion*: Overall analysis demonstrated an increased incidence of delirium in older females with greater comorbid conditions. It was also found to be associated with increased morbidity and mortality. We recommend clinicians put greater effort into recognising risk factors of delirium and diagnosing it in a timely manner to mitigate its effects.

## Introduction

Postoperative delirium is a common complication of surgical interventions in the elderly. Characterised by acute and fluctuating impairment of cognition, attention, and consciousness, it is a multifactorial condition that is associated with poor outcomes. Although its incidence is substantially influenced by patient-related risk factors, previous reviews have indicated that it affects a large proportion of orthopaedic patients. In 2007, a meta-analysis of 26 studies reported higher rates of delirium in patients undergoing hip fracture surgery (ranging from 4 to 53 percent) compared with elective surgery (3.6–28.3 percent) [1]. Delirium has immediate adverse implications for the orthopaedic patient such as an increased risk of major complications, slow recovery and therefore a longer inpatient stay. Patients are typically less compliant with rehabilitation protocols and are more likely to require nursing home placement after discharge. In the longer term, it is also associated with the development of dementia [2]. As a preventable condition in 30–40% of cases [3] delirium holds substantial public health relevance as a target for interventions to prevent its associated burden of downstream complications and costs [4].

Causes of postoperative delirium in patients with hip fracture are complex and not well understood [5]. Its onset is thought to reflect an accumulation of predisposing patient risk factors and the physiological stress of surgery [6]. Thus, identifying at risk individuals is critical to creating prevention and treatment pathways. The purpose of this prospective observational study was to determine the commonest risk factors for new onset delirium after hip fracture surgery in the UK, to establish its incidence, and associated mortality.

## Materials and methods

In this single-center, prospective, cohort study we enrolled all patients presenting to our institution with a neck of femur (NOF) fracture between 2015 and 2016. Our primary objective was to evaluate the incidence and predisposing risk factors for developing delirium after NOF fracture surgery. We compared patients with new-onset post-operative delirium to those who did not develop delirium using multiple variables at each stage of their treatment pathway. For purpose of analysis the following data were extracted: patient's age and gender; date of injury; co-morbidities; place of residence before admission; cognitive function at the time of admission; time to surgery; type of anaesthesia and procedure performed. Other variables studied in the postoperative period were presence of anaemia (Hb < 100 g/L), new onset hypoxaemia, significant hypotension, renal failure, and date of death if applicable. Patient records were reviewed at 1-year to assess rate of mortality.

### Setting

Our institution is a busy acute hospital with 470 beds serving a population of around 290 000. There is a well-established NOF fracture treatment pathway which aims for prompt haemodynamic resuscitation, early clinical assessment, radiological diagnosis and definitive management within 36 h of attendance. Patients are offered prompt and appropriate analgesia (which may include an ilio-fasical block in addition to oral analgesics) in the emergency department. Patients are then admitted to a dedicated orthopaedic ward for pre-operative optimisation, surgical and orthogeriatric assessment. Surgery is performed in line with national guidance preferring cemented hemiarthroplasty (or total hip arthroplasty) in patients with a displaced intracapsular fracture. Extracapsular fractures are treated using dynamic hip screw fixation or cephalo-medullary devices in most cases. Where appropriate, patients are offered a choice of spinal or general anaesthesia after assessment by the consultant anaesthesist. Postoperative rehabilitation protocols were similar in all patients. Patients were routinely seen by the physiotherapists and occupational therapists as of the first postoperative day. Discharge destination post-surgery varies from rehabilitation wards within the trust, intermediate care centres to home with a safe package of care. The study was approved by our institutional review board.

### Diagnosis of delirium

A ten-point abbreviated mental test (AMT) assessment was performed at the time of admission to assess baseline cognitive function. Patients were then assessed daily on ward rounds by a senior orthogeriatric physician. Any change in consciousness and/or cognition reported in the physician or nursing notes was recorded. Presence of delirium was confirmed using the Diagnostic and Statistical Manual of Mental Disorders (DSM 4th Edition) criteria (Table 1) [7].

**Table 1:** Diagnostic criteria for delirium according to DSM-IV [7].

A	Disturbance of consciousness (i.e. reduced clarity of awareness of the environment) with reduced ability to focus, sustain, or shift attention.
B	A change in cognition (such as memory deficit, disorientation, language disturbance) or the development of a perceptual disturbance that is not better accounted for by a pre-existing, established, or evolving dementia.
C	The disturbance develops over a short period of time (usually hours to days) and tends to fluctuate during course of the day.
D	There is evidence from the history, physical examination, or laboratory findings that the disturbance is caused by the direct physiological consequences of a general medical condition.

### Data analysis

Statistical analysis was performed using SPSS version 20 (IBM; Armonk, NY). Significant differences between groups were identified by independent *t* tests for continuous variables and *χ*^2^ tests for categorical variables. Binary logistic regression using the stepwise method was performed to determine independent risk factors for postoperative delirium. *p* values <0.05 were considered statistically significant.

## Results

During the 12-month study period, 395 patients underwent surgery for NOF fracture at our institution. Fourteen patients were excluded from analysis due to missing data. Of the remaining 381 patients, 70 patients (18%) developed new-onset delirium post-operatively. Patients experiencing delirium were significantly older (average age 83 vs. 78, *p* = 0.019) and more likely to be female (79% vs. 67%, *p* = 0.062) than non-affected patients (Table 2). Patients who already had a diagnosis of dementia on admission were significantly more likely to develop post-operative delirium compared with those with no cognitive dysfunction on admission (51% vs. 37% respectively, *p* = 0.02).

**Table 2:** Patient demographics.

	Non-delirious	Delirious	*p*
Total number of patients	311 (82%)	70 (18%)	–
Average age (median year)	78	83	0.019
<25 years	3 (100%)	0	–
25–45 years	5 (100%)	0	–
46–64 years	37 (95%)	2 (5%)	–
65–74 years	42 (83%)	9 (17%)	–
≥75 years	224 (79%)	59 (21%)	–
Sex (% female)	67.2	78.6	0.062
Nottingham hip fracture score (median [IQR])	5 [3–6]	5 [5–6]	0.071
Pre-existing dementia	115 (37%)	36 (51%)	0.026

The majority of patients were treated with either a dynamic hip screw (47%) or a hemiarthroplasty (30%) with no significant differences in choice of treatment between the two groups (Table 3).

**Table 3:** Treatment modality.

	Non-delirious (*n*)	Delirious (*n*)	*p*
Dynamic hip screw	152 (82%)	33 (18%)	0.793
Hemiarthroplasty	91 (76%)	28 (24%)	0.08
Total Hip Replacement	20 (95%)	1 (5%)	0.098
Intramedullary Nail	45 (87%)	7 (13%)	0.325

**Figure 1:**
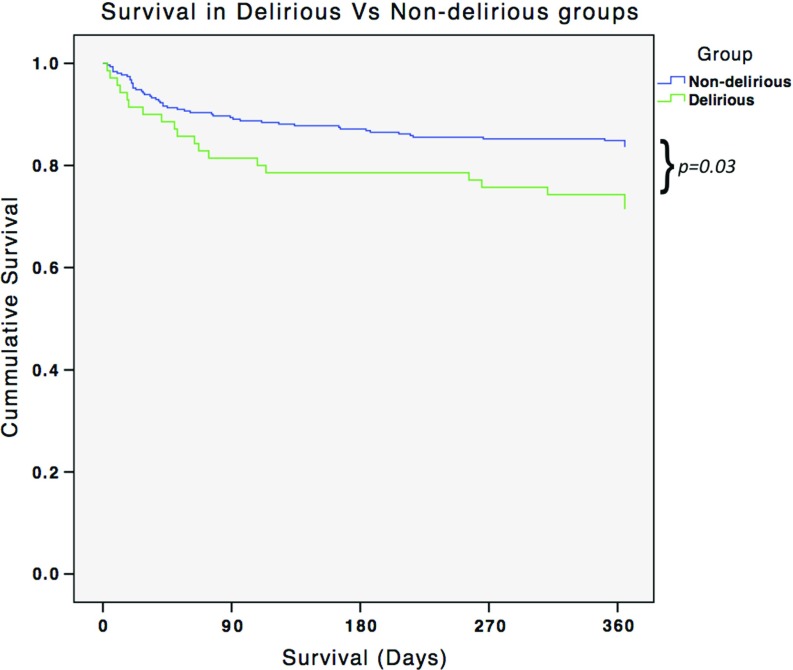
Kaplan Meier plot.

Presence of delirium was associated with significantly increased length of stay (average Length of stay 13 days vs. 10 days, *p* = 0.001. Incidence of mortality at 1 year was significantly higher in patients with a diagnosis of postoperative delirium (25.7%) compared to patients without delirium (15%) *p* = 0.026 (Figure 1). Mortality within 30-days followed the same trend (10% vs. 6%) however did not reach statistical significance (*p* = 0.243) (Table 4).

**Table 4:** Mortality.

	Non-delirious	Delirious	*p*
30 days	6%	10%	0.24
1 year	15%	25.7%	0.03

Logistic regression analysis identified 7 independent predictors for postoperative delirium (Table 5). These included age ≥65 years (OR 5.8, CI 1.4–24.3), presence of anaemia (OR 2.9, CI 1.5–5.5), new onset hypoxia (OR 2.86, CI 1.4–5.8), cardiac disease (OR 2.8, CI 1.5–5), COPD (OR 2.5, CI 1.5–4.2), new onset electrolyte imbalance (OR 2.2 CI 1.2–3.7) and renal failure (OR 1.9, CI 1.1–3.3). Factors which did not show statistically significant association with the development of delirium included delay in surgery i.e. >36 h from admission to surgery (OR 0.98, CI 0.5–1.8), use of general anaesthetic (OR 1.2, CI 0.5–2.6), prolonged operating time i.e. >2 h (OR 1.3, CI 0.6–2.7), and post-operative hypotension i.e. >30% from baseline (OR 0.9, CI 0.4–2.0).

**Table 5:** Risk factors for postoperative delirium.

	Odds ratio	95% Confidence interval		*p*
Age ≥ 65	5.75	1.36	24.3	0.017
Hb <100	2.91	1.53	5.54	<0.001
New onset hypoxaemia	2.86	1.4	5.84	0.004
Cardiac disease	2.76	1.52	5.04	0.001
Renal failure	1.92	1.13	3.27	0.016
COPD	2.47	1.46	4.18	0.001
New onset electrolyte imbalance	2.15	1.23	3.75	0.007
>36 h from admission to surgery	0.98	0.53	1.82	0.95
Use of general anaesthetic	1.18	0.53	2.64	0.692

## Discussion

Neck of femur fractures are a major public health concern due to the increasing ageing population. Majority of fractures occur in patients over the age of 65, frequently in those with frailty and dependency, and with pre-existing medical problems. For these reasons, it provides a good clinical model to study and understand delirium. This study focused on incident (or new onset) delirium which occurred exclusively in the postoperative period. We identified a number of independent risk factors that correlate with the onset of post-operative delirium in NOF fracture patients: increasing age; post-operative anaemia; cardiorespiratory co-morbidities, and acute electrolyte imbalance. Identifying patients at increased risk of delirium after surgery could enable pre-emptive action to mitigate this risk.

Since the implementation of National guidelines on hip fracture management and severe financial penalties for healthcare trusts that fail to comply with efficiency targets, most institutions, including ours, have well-defined treatment pathways to facilitate early surgery and targeted rehabilitation [8,9]. Moreover, orthogeriatrician-led perioperative care has led to a notable reduction in morbidity and mortality amongst hip fracture patients. In our study, we found a much lower incidence of delirium than that found in previous studies [10–12]. This finding may in part be explained by the aforementioned improvements in care but there are several other possible explanations including variations in definition of delirium and diagnostic accuracy. Because delirium is by nature a fluctuant disorder, which may sometimes last for a short duration or in its hypoactive form, it is possible that some cases may have been missed.

Previous reports have associated post-operative delirium with increased length of hospital stay, rate of discharge to a nursing home, health and social care costs [13,14]. Similar findings were observed in this study. Presence of delirium on average amounted to 3 extra days of hospital stay. Although cost analysis was beyond the scope of our study, based on recently published figures, it is estimated that a district general hospital in the NHS would incur an additional £894 per patient for 3 days of inpatient orthopaedic or rehabilitative care [15]. This estimate excludes the cost of any additional physiotherapy or occupational therapy sessions, additional nursing procedures or ward rounds, and the cost of treating delirium itself. Finally, adding on costs for potential post discharge dependency, such as nursing home care, the financial burden of postoperative delirium can rise even higher.

Controversy exists regarding whether postoperative delirium is an independent predictor of mortality [16,17]. In our study, about one-quarter of patients who developed delirium died within 1 year, which was significantly higher than non-delirious patients. However, given the high prevalence of coexisting frailty and morbidity in these patients, a direct causal relationship cannot be determined. Indeed, dementia, COPD, chest infection, heart failure, anaemia, electrolyte disturbance, elevated urea/creatinine, and malignancy, have all been described as risk factors for increased mortality in the months following a hip fracture [18]. An important limitation of our study was that prospective data on patients' health and function was only recorded until the time of discharge. Other factors that may have contributed to mortalities that occurred following discharge from hospital, remain largely unknown.

Delirium occurs as a consequence of a combination of predisposing, non-modifiable factors--such as age, co-existing medical illnesses etc.--and precipitating factors which are often modifiable. The risk factors identified through logistic regression in our study are generally in agreement with previous reports on postoperative delirium. Elderly patients are considered to be at higher risk because many of the aforementioned predisposing risk factors accumulate and overlap with ageing. For this reason, delirium was most frequently observed amongst the octogenarians. However, through binary logistic regression analysis, patients as young as 65 years of age were found to have a significantly increased risk of developing delirium compared to those younger than 65 years. Although predictors such as age are unmodifiable, they should be used in stratifying high risk patients on admission so that preventative strategies can be better targeted towards those at high risk. Modifiable risk factors such as anaemia and hypoxia can be prevented or treated to avoid prolonged cerebral hypoxaemia, which is thought to be an important underlying phenomenon in the development of delirium [19,20]. A number of previous studies investigating the role of anaemia and blood transfusion within the prevention and treatment strategies for delirium have also found low hemoglobin level to be associated with delirium, and receiving a blood transfusion to be associated with a lower delirium incidence [21]. Similarly, supplemental oxygen (3–4 L/min) continually till day 2 post-surgery, or while patient's oxygen saturation is not ≥ 95% without oxygen, have proven to reduce delirium risk [22,23]. Based on previous systematic reviews, the Association of Anaesthetists of Great Britain and Ireland (AAGBI) and Scottish Intercollegiate Guidelines Network (SIGN) recommend the use of regional anaesthesia with the aim of avoiding general anaesthesia and opioid drugs in all patients unless contraindicated [7,8,24]. In this study, general anaesthesia was not associated with an increased risk of developing post-operative delirium. A more up-to-date systematic review is currently underway evaluating both experimental and observational evidence on the use of regional compared to general anaesthesia in patients undergoing hip fracture surgery, with a focus on delirium as an outcome [25]. Because delirium is considered a multifactorial condition, addressing a single risk factor is unlikely to be successful, therefore multicomponent approaches will be most effective for both prevention and treatment.

Despite significant improvements in both surgery and rehabilitation in recent decades, hip fracture remains, for patients and their carers, a much-feared injury. Because the onset of delirium signals underlying ill health, a comprehensive multidisciplinary approach is required in treatment and indeed prevention of delirium. A clear understanding of risk factors for postoperative delirium helps in the selection of individuals who might benefit from targeted perioperative intervention.

## Conflict of interest

The authors declare that they have no conflict of interest.
